# DCN-MAC: A Dynamic Channel Negotiation MAC Mechanism for Underwater Acoustic Sensor Networks

**DOI:** 10.3390/s20020406

**Published:** 2020-01-10

**Authors:** Yishan Su, Lijie Dong, Qiuling Yang

**Affiliations:** 1 School of Electrical and Information Engineering, Tianjin University, Tianjin 300072, China; yishan.su@tju.edu.cn (Y.S.); lijay_dong@tju.edu.cn (L.D.); 2 School of Computer Science & Cyberspace Security, Hainan University, Haikou 570228, China

**Keywords:** underwater acoustic sensor networks, media access control (MAC), spatial–temporal mapping

## Abstract

In the design of media access control (MAC) mechanism in underwater acoustic sensor networks (UASNs), due to the propagation characteristic of low-speed underwater acoustic signals, it is necessary to solve the spatial–temporal uncertainty problem. In order to avoid the multi-user access conflict in underwater networks, reduce the energy cost and improve the throughput and fairness across the network, a dynamic channel negotiation MAC mechanism based on spatial–temporal mapping of receiving queue (DCN-MAC) was proposed. DCN-MAC uses a duty cycle mechanism and implements a network management based on dynamic single node wake-up. The awakening node collects the request to send (RTS) and network status information in the network to solve the problem of space-temporal uncertainty and the highly dynamic needs of network access nodes and access services. The simulation results show that in different network scenarios, especially in those featuring high density and heavy network load, compared with the traditional underwater acoustic network MAC protocols, this protocol can effectively improve the network throughput and reduce the packet loss probability caused by multi-node conflict.

## 1. Introduction

Underwater acoustic sensor networks (UASNs) are one of the most effective tools for exploring and observing the ocean. It has a wide range of application scenarios: marine pollution monitoring, marine resource exploration, military observation, earthquake disaster monitoring, and so on [1,2]. Therefore, the research of related protocols around UASNs has a sanguine prospect for wide application. UASNs deploys sensor nodes of underwater acoustic communicator in complex underwater environment to collect and aggregate the data, and finally transmits data to shore base station through the relay of buoy nodes for further processing [3]. Underwater acoustic communication modem uses acoustic wave as an information carrier to realize underwater wireless communication. The reason why we use an acoustic wave as the information carrier is that electromagnetic wave has a serious propagation loss in water and is unable to achieve reliable communication over long distances. Although acoustic waves can be used as an information carrier to achieve more reliable and long-distance wireless communication than electromagnetic wave, underwater acoustic communication still faces the situation of lower speed, delay, and larger error code due to the serious multipath interference and Doppler interference of underwater acoustic channel [4,5], limited available bandwidth, and only about 1500 m/s of sound speed (five orders of magnitude lower than the velocity of electromagnetic wave). This shortage of underwater acoustic communication leads to many problems of UASNs which are different from terrestrial wireless sensor networks, including variable packet propagation delay, high energy consumption, limited channel resources [6,7], etc.

**Figure 1:**
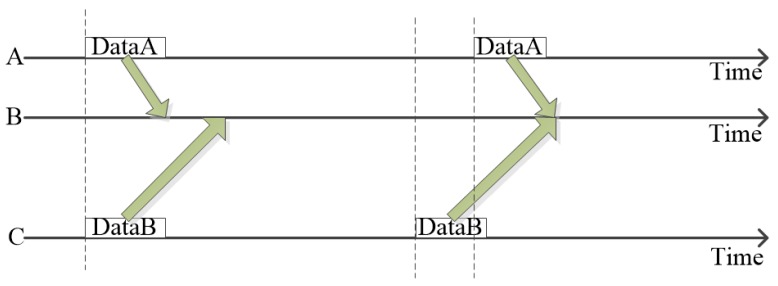
Spatial–temporal uncertainty.

Media access control (MAC) protocol is an important component of UASNs. The main function of underwater MAC protocol is to achieve the rational distribution of underwater acoustic channels, so that multiple underwater nodes can share the same channel without conflict, maximize the throughput of the network, save energy and make sure the fairness between nodes [8,9]. However, due to the properties of underwater acoustic channel, underwater MAC protocol faces such problems as large round trip delay, dynamic node moving, high energy consumption and high communication error rate. Among them, the large round trip delay of data packets leads to the spatial–temporal uncertainty in underwater MAC protocol. Therefore the scheduling problem of information transmission is a spatial–temporal problem [8,10]. Figure 1 shows the problem of spatial–temporal uncertainty. In the first case, nodes A and C send their data packets at the same time. However the distance between AB and BC is different, so the data packets of nodes A and C will not reach node B at the same time, and there will be no conflict. But in the second case, node A sends later than node C. Conflicting occurs due to the different propagation delay between node A and node C. This phenomenon is called spatial–temporal uncertainty. Similar to land-based sensor networks, the design of underwater MAC protocol also faces the dynamic adjustment of network structure caused by the burst of node data transmission service [11]. In each data transmission, different transmission nodes mean different network structure. Usually there are multiple sending nodes in the network, and each node randomly generates data sending services, which results in different network topologies to be processed at each stage.If the protocol can only be applied to a specific network topology, channel resources will be extremely wasted. It requires that the protocol can be flexibly adapted to the dynamic adjustment of network structure and avoid the waste of channel resources caused by the burst of data.

In order to solve the above problems, some reservation-based MAC protocols dedicated to UASNs have been proposed. These protocols use TDMA [12] or CDMA [13] to partition channel slots or chips, and then combine the information of the nodes to achieve channel resource allocation. Therefore these reservation-based methods have a strong collision avoidance capability. However, such reservation-based protocols are often not adapt to the dynamic adjustment of the network, including the dynamic traffic and the dynamic topology of nodes in the network. Although some protocols, such as ED-MAC [14], add a sub-slot mechanism to ensure that newly joined nodes access the network, there is still consumption of sub-slot resources for dynamic burst traffic conditions between multiple nodes. In comparison, contention-based MAC protocols based on random access or handshake mechanisms are more suitable for network dynamics. Such as the handshake-based FAMA [15] protocol makes reservations for channels through control packets request to send (RTS) and clear to send (CTS), which fully reduces the possibility of data packet collisions, and can still ensure the full occupation of the channel during node business bursts and network structure adjustment. However, most handshake-based protocols do not consider the problem of space-time uncertainty, and the frequent interaction of control packets not only reduces the channel utilization but also consumes a lot of energy, and the nodes stay in the listening state for a long time will also accelerate the energy consumption.

In this paper, we propose a dynamic channel network MAC (DCN-MAC) protocol for data aggregation and intra-cluster nodes to access the scenarios scheduling. DCN-MAC uses the duty cycle mechanism to determine the sending time of each node according to the location information of different nodes and the size of the transmitted data packet, eliminating the problem of space-time uncertainty, allowing all data to reach the receiving node according to the data queue, and avoiding data packets collision. DCN-MAC only considers the sending requests of nodes in this cycle. Therefore, it can flexibly adapt to the burstiness of node services and the dynamic structure of the network, so the protocol has a strong dynamic adaptability. In the reservation phase, we use the conflict avoidance mechanism based on the game theory, which regards multi-node simultaneously wake up the contention channel as a non-cooperative incomplete information game. This game theory can be solved by Nash equilibrium equation, that can effectively deal with the simultaneous wake-up of multi-node. The simulation results show that the protocol can effectively improve the channel utilization and optimize the throughput performance of the network.

Section 1 introduces the application background of UASNs protocols and the main challenges of designing underwater acoustic MAC protocol. Section 2 introduces the related work of MAC protocol for UASNs. Section 3 introduces the DCN-MAC protocol in detail. Section 4 shows the simulation results of DCN-MAC protocol to verify the performance. Section 5 is about the conclusion of study and prospect for the further study.

## 2. Related Work

The function of MAC protocol in UASNs is to allocate underwater acoustic channel reasonably and fairly to avoid data conflict between nodes. According to the different channel allocation mechanism of underwater MAC protocol, it can be divided into two types: contention-based and contention-free.

The contention-free MAC protocol usually uses scheduling or nodes clustering to avoid conflict. Traditional contention-free conflict avoidance mechanism includes TDMA [12], CDMA [13] and so on.This type of protocol avoids conflicts between nodes through fixed allocation of time slots or chips. Because the limited bandwidth and large round trip delay of underwater acoustic channel, these methods can not be directly applied to underwater MAC protocol. Therefore, CB-TDMAMAC [16] protocol based on TDMA and nodes clustering is proposed to establish spatial–temporal conflict table to achieve the effect of conflict avoidance. In this protocol, cluster-heads are selected through contend between the nodes firstly. After that, other nodes are scheduled by the cluster-heads, so that the nodes outside the communication radius of the other party can send at the same time. GC-MAC [17] is a protocol based on graph theory for contention-free conflict avoidance. This protocol performs unified scheduling for all nodes based on the known three-dimensional location information of all nodes in the network. Nodes outside the communication radius of a node are identified as non-conflict nodes and marked with the same color. Then finish the channel time slot assignment, and the nodes with the same color can send data in the same time slot to achieve non-conflict data transmission. Although these protocols are conflict-free, they all treat nodes beyond the communication radius as conflict-free nodes, so they are not suitable for dense distribution of nodes in the network. Both of them need to know precisely the three-dimensional location information of nodes, and pre-allocate resources according to network topology or the number of nodes. Therefore, they are not suitable for dynamic adjustment of networks, and underwater network location is still a serious challenge.

The contention-based MAC protocol can be further divided into two mechanisms: handshake or random sending. ALOHA protocol and its derivatives are the most common random transmission mechanism. In this kind of mechanism, a node sends a short control packet RTS directly after it has a data transmission service. But if a control packet from another node is already received, the avoidance algorithm will run and wait to be sent. For example, T-Lohi [18] is a random transmission mechanism protocol. In addition to a half-duplex communication modem, each node in the protocol is equipped with a low-power tone signal receiver. A node will send a tone signal to the contention channel after having a data transmission service. Then the node monitors the channel through low consumption receiver, and runs the contention detection and counting algorithm. In order to count the number of nodes that contend for the channel in the same time slot. T-Lohi optimized the contention avoidance algorithm between nodes based on random intervention. However, because the protocol still uses avoidance algorithm when dealing with multi-node conflict, the probability of avoidance increases rapidly when the network load increases, which leads to the performance degradation of the protocol.

The most widely used MAC protocol based on the handshake mechanism is slotted FAMA [19] protocol. This protocol achieves conflict avoidance and the channel allocation through control packet interaction between nodes. However since each control packet needs to wait for a maximum propagation delay, so the channel utilization is low. When the network load is increased, there will be a large number of conflicts resulting in throughput degradation due to the lack of optimizing the avoidance algorithm. Shortly after, two handshake-based MAC protocols, DRAMAC [20] and SF-MAC [21], were proposed. DRAMAC uses the method of multi-channel to improve network throughput.The channel is divided into a control channel and multiple data channels. Nodes determine the sub-channel through the control channel interaction report length and network load. SF-MAC optimizes the fairness performance of the network by calculating the distance between the sending node and the receiving node.The receiving node first collects all the sending request packets, and determines the optimal sending node according to the distance between different nodes and considers its transmission time. However, the channel resources are not fully utilized due to the multiple handshakes of the control packet and the waiting time after the control packet is sent. RIPT [22] is a combination of handshake mechanism and MAC protocol initiated by the receiver. It is the first to apply the concept of data queue to UASNs. The protocol starts a data transmission by sending control packets at the receiver, collects the information from the sending node, determines the sending time of each sending node, and makes the data arrive at the receiving node by queue, so as to receive the data without conflict. MR-MAC [23] is an improvement of RIPT. It can receive data from multiple receiving nodes in a single scheduling, and make sure the data arrive at the receiving node by queue. The two data queue-based protocols mentioned above reducing the probability of data conflict in the network, by scheduling the receiving node to the sending node. It does not take into account the conflict problems in negotiations between nodes and the spatial–temporal uncertainty caused by the different location of nodes. Moreover, all nodes are continuously monitoring the channel, which cause a large energy loss.

## 3. DCN-MAC Protocol

### 3.1. DCN-MAC Overview

**Figure 2:**
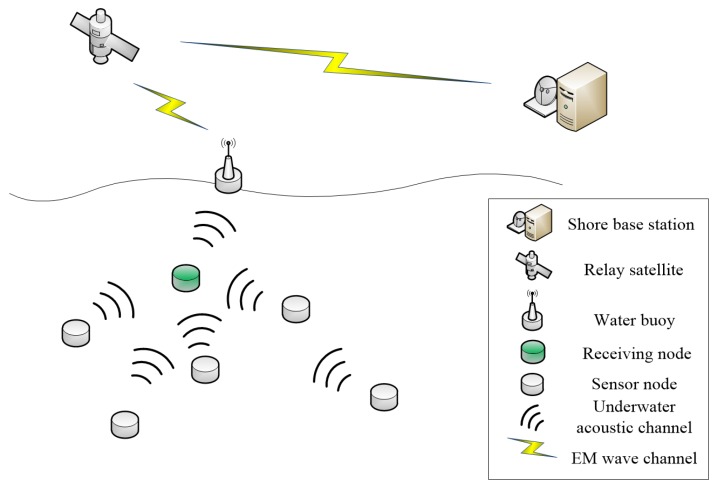
Network model.

DCN-MAC is a MAC protocol for data aggregation of underwater sensor nodes. Figure 2 shows a UASN consisting of multiple underwater sensor nodes and a surface buoy. Each node is equipped with an underwater acoustic communication modem. The receiving node is responsible for collecting sensor data from other nodes and forwarding them to the buoy on the water surface. After that, the underwater sensor data is transmitted to the shore base station through satellite communication for processing. Unlike the traditional MAC protocols based on the channel reservation allocation, DCN-MAC protocol is based on negotiation to implements channel allocation, and determines the sending time of the node to be sent by the scheduling algorithm. It eliminates the influence of spatial–temporal uncertainty in UASNs, and makes all data arrive at the receiving node in form of data queues to realize conflict-free data transmission. Moreover, the DCN-MAC protocol does not need the node to remain awake all the time to save energy. In the protocol, when multi-nodes are awakened at the same time to contend for channels, it is regarded as a non-cooperative incomplete information game. It is solved by Nash equilibrium equation, and random collision of data packets caused by simultaneous wake-up and transmission of multi-node is reduced.

This protocol is applied to schedule the accessing of intra-cluster nodes in a single-hop quasi-static network or clustering network with data aggregation [24]. In the network initialization stage, the synchronization of the network is achieved by the time information broadcast between the nodes [25]. After initialization, the receiving node will wake up and sleep periodically. According to the sleep and wake-up states of receiving nodes, the network is divided into two stages: channel reservation and data transmission. They finish the determination of node sending time and data transmission respectively.

### 3.2. The Stage of Channel Reservation

At this stage, the scheduling node is in a dormant state. The sending node wakes up if it has a data transmission service. After that, each awakening node completes channel reservation allocation through control packet interaction. By combining the location of the node to be sent, the length of the data packet and residual energy information, a data sending table (ST) is established. Until the receiving node wakes up and enters the stage of data transmission, then each node starts its own data transmission according to the appointed time in the schedule. In the following part, the channels reserve process, the sending time algorithm based on data queues receiving and the conflict avoidance mechanism based on the game theory will be introduced in detail.

#### 3.2.1. Channel Allocation Process

**Figure 3:**
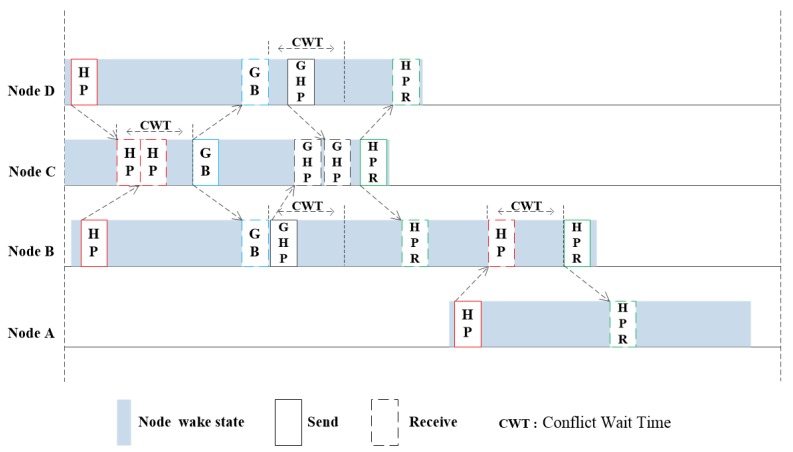
Channel contention game process.

At this part, the process of channel reservation is introduced in detail. There is a wake-up node scheduling node (SN) in the network, and the remaining nodes are dormant if there is no data transfer. SN is not a specific node, but will choose different nodes as SN over time. When the sending node in the network has a data transmission service, the sleep state will be ended immediately. It broadcasts hello packet (HP) to inform that SN in the current network has a new data request schedule. HP contains the ID of the node, the length of the data packet, the remaining energy, and the propagation delay to the receiving node. If a node waits for request wait time (RWT, which equals twice the maximum propagation delay of the network) after sending HP and does not receive a response from SN, then there is no SN in the network, and the node remains awake as SN. When there is no SN in the network, if multiple nodes wake up at the same time, these nodes will collect HP messages of other wake-up nodes, compare the remaining energy information of each node, and the node with the highest remaining energy remains as the SN to stay awake. SN will enter conflict wait time (CWT) state after receiving an HP message. The size of CWT equals to the maximum propagation delay in the network. If only one HP message is received during the CWT time, there is no contention among multi-node in this scheduling. SN runs the ST algorithm and determines the ID of the next SN based on the remaining energy information in HP message. Next, it calculates the sending time of non-scheduling node, and broadcasts the data into the hello packet reply (HPR) message to notify the sending node. For example, Figure 3 shows the channel reservation of node A. Node A wakes up and sends HP message. At this time, scheduling node B in the network receives HP message from node A. After comparing the residual energy information of nodes A and B, it is determined that node A will act as SN in the following time, and running ST scheduling algorithm to calculate the sending time of node B. Then write it into the sending table, and fill the scheduling results into the HPR message to reply node A, then node B begins to sleep, node A remains awake as SN.

If there are multi-node waking up in the adjacent time, it is necessary to run the contention scheduling mechanism to solve the conflict problem. The channel reservation process of multi-node conflicts are shown in the scheduling process of nodes B and D in Figure 3. Node B and node D wake up almost at the same time, and the HP messages sent by both nodes reach scheduling node C within CWT (the length of CWT is equal to the maximum propagation delay in the network). Therefore, node C runs the conflict avoidance algorithm. During the conflict waiting time, the number of nodes in conflict at this stage is 2. At the end of CWT, a contention acknowledgement frame is sent, which contains the number of conflict nodes in this stage. After receiving the game begin (GB, which includes the number of nodes participating in the current contention channel) message, node B and D determine their sending probability according to the number (N) of contention nodes contained in it. Then, send game hello packet (GHB, which contains the same information as HP) message in game time (GT, the size of which is equal to the maximum propagation delay of the network) according to the optimal strategy, and transmit its own information to SN. GHB messages of nodes C and D arrive at node C in different times, and then node C compares the residual energy with nodes B and D. Node B is used as SN to stay awake, the sending time of node C and D is filled in the sending table and then passed to node B. The algorithm process pseudocodes of ordinary sending node and the scheduling node are respectively shown in Algorithm 1 and Algorithm 2:

**Algorithm 1** Algorithm of the sending node **for** each node(i) wakes up **do**  broadcast HP  **if**
T>RWT&&noHPR  //T is time after this node broadcasts HP   node(i) = SN  **else if** receive HPR   **if** node(i).re > SN.re    node(i) = SN   **else**    get node(i).starttime and go to Sleep   **end if**  **end if** **end for**

**Algorithm 2** Algorithm of the scheduling node **if** SN receives HP  **if**
T>RWT&&NUM>1  //NUM is the number of sending node   broadcast GB  **else**   broadcast HPR  **end if** **end if**

#### 3.2.2. Scheduling Algorithm of Sending Time Based on Data Queues

The main purpose of scheduling algorithm is to eliminate the influence of spatial–temporal uncertainty on channel resource allocation. Due to the long propagation delay of underwater acoustic channel, node A may send first. However, because the propagation delay of node A arriving at the scheduling node is long, the receiving node will be idle during this propagation delay. If other nodes are closer to the Scheduling node, they can receive the data of this node in this period of idle time. As long as the parts filled by each sending node do not coincide, there will be no conflict at the receiving node. In this way, the waste of channel resources caused by the spatial–temporal uncertainty can be reduced. The scheduling Algorithm 3 is implemented by SN, which keeps awake in the network. When SN receives HP or GHB message from other nodes, it first compares the remaining energy of two or more nodes, selects the node that continues to wake up as SN, and then runs the scheduling algorithm to calculate the sending time of all other non-scheduling nodes.

The time of each node sending data is based on the existing node sending time in ST and the propagation delay from the corresponding node to the scheduling node, combined with its own information to calculate its own sending time. Therefore, the sending time of the first scheduled node is ST1 = 0, which means that the node starts sending data immediately after the receiving node wakes up. Other nodes have saved the sending time in the sending table before being scheduled, so it is necessary to consider the time period of receiving node that other nodes will occupy when sending data, and calculate whether the length of their data packets can be inserted in the existing time period to avoid the conflict of data packets. Assuming that the packet length of node C is less than the length of the data packet occupation period between two nodes, and node C can arrive in this time period, then the sending time of node C is STC = STA + delayA + datalen – delayC. The data packets of node C are received between node A and node B by using the scheduling algorithm. The sending time determination steps of each newly scheduled node X is as follows. ST already contains the sending time information of multi-node and the number of data packets of these nodes.

**Algorithm 3** Scheduling algorithm of sending time X.starttime = 0 //node(i).starttime is the time that node(i) begin to send X.endtime = X.starttime +X.pl× 0.17+X.delay //node(i).pl is the data packet length of node(i) //node(i).delay is the propagation delay to the receiving node **for**
i=1:N  **if**
X.endtime<node(i).starttime   break  **else**   X.starttime = node(i).endtime-X.pl*0.17+X.delay   X.endtime =X.starttime+X.pl*0.17+X.delay  **end if** **end for**

#### 3.2.3. Conflict Avoidance Mechanism Based on Game Theory

Because each sending node wakes up randomly, there will be multi-node waking up at the same time, which will lead to conflict at the scheduling node. In the multi-node conflict, the conflict node itself does not know how many nodes are in conflict at this time. Moreover, due to the high energy consumption and long propagation delay of underwater acoustic communication, it is difficult to broadcast the strategy selected by the node itself to other nodes, so the node itself knows nothing about the other nodes. In the contention, each node can be regarded as a rational and selfish user, hoping to obtain the right to use the channel and occupy it for a long time, so it can be regarded as a non-cooperative incomplete information game.

Firstly, the payoff of the game is defined. In order to solve the problem of multi-node conflict, reduce the energy loss caused by the conflict, and improve the utilization rate of underwater acoustic channel. Therefore, the utility of the game mainly considers the overall energy consumption of the network and the utilization rate of the channel. The following is an analysis of the state in which only two nodes j and i contend. In the game between two nodes, there are two strategies that nodes can choose: waiting and sending. The payoff of two nodes contention channel is shown in Table 1. Value of failure (VF) represents the payoff of the node in case of conflict, value of wait (VW) represents the payoff of the node avoidance in this competition, and value of send (VS) represents the payoff of sending successfully. The channel resource and node energy are wasted after the node conflict, so VF has the least payoff. VW indicates that the node does not consume energy but the channel resources are still wasted, *VS* indicates that the channel and node energy are effectively used, so we can get VS>VW>VF.

Obviously, when one node chooses to send and the other node avoidance, the overall payoff is the highest. Therefore, there are two optimal equilibrium strategies in this game, called nash equilibrium. Thus the optimal strategy combination of node i and node j is: (Si,Wj) or (Wi,Sj). By simplifying the node payoff in Table 1, Table 2 is obtained., where *e* represents the energy consumed by sending data packets:

If both nodes choose to send, the network will have the lowest payoff, because each node loses energy. If both nodes choose to wait, the payoff of each node is 0. Therefore, when node j chooses to send, the best strategy for node i is to choose to wait, but node i don’t know the strategy chosen by node j. Therefore, the best choice at this time is that the other two nodes choose to send with a 50% probability, which will minimize the probability of conflict and maximize the possible payoff.

Then start modeling and solving the game. First of all, confirm that the purpose of this game is to enable each node in conflict can send its own GHB message to SN. The GHB message contains the information of each node, so there is no need to worry about the order in which the GHB packets arrive at SN. Nodes can send GHB at any time during the contention gaming period. As long as it can reach SN, it will not affect the scheduling result. Therefore, it is the most effective way to reduce the possibility of conflict by sending GHB with a uniformly distributed probability during the contention period. At this time, game solution is to find out the best transmission probability. There are only two kinds of strategies that nodes can choose are waiting or sending. Therefore, the node has a finite set of opposite strategies: *S ={ Ss,Sw}*, where *Ss* means that the node sends GHB, *Sw* means that the node waits for sending. The probability of selecting two strategies is *P^S^* and *P^W^* respectively. Set the total payoff of the network is *W*. According to the different node selection strategies, calculate W:

(1) $$W=P_{i}^{S}P_{j}^{S}\left(V_{i}^{F}+V_{j}^{F}\right)+P_{i}^{W}P_{j}^{S}\left(V_{i}^{W}+V_{j}^{S}\right)+P_{i}^{S}P_{j}^{W}\left(V_{i}^{S}+V_{j}^{W}\right)+P_{i}^{W}P_{j}^{W}\left(V_{i}^{W}+V_{j}^{W}\right)$$

Each node in the network is the same, so the benefits of each node are the same under the same circumstances, so the following formula can be obtained.

(2) $$\left\{\begin{array}{c} V_{i}^{F}=V_{j}^{F}\\ V_{i}^{W}=V_{j}^{W}\\ V_{i}^{S}=V_{j}^{S} \end{array}\right.$$

(3) $$\left\{\begin{array}{c} P_{i}^{S}+P_{i}^{W}=1\\ P_{j}^{S}+P_{j}^{W}=1 \end{array}\right.$$

plug (2) and (3) into (1) to get the formula:

(4) $$W=2P_{i}P_{j}V^{F}+\left(V^{W}+V^{S}\right)\left(P_{i}+P_{j}-2P_{i}P_{j}\right)+2\left(1-P_{i}\right)\left(1-P_{j}\right)V^{W}$$

To explore the partial derivative of W, we can draw a formula:

(5) $$\frac{\partial W}{\partial P_{i}}=0,\frac{\partial W}{\partial P_{j}}=0$$

Then, draw:

(6) $$P_{i}=P_{j}=\frac{V^{W}-V^{S}}{2(V^{F}-V^{S})}$$

The best solution to the conflict between two nodes is shown in formula 6.Nodes send their own GHB messages during the gaming contention according to the uniform distribution.

In terms of the games among N nodes, every node has the same single-stage utility value (VF, VW, VS).

In addition, it is assumed that the nodes send the same kind of traffic have a symmetric transmission probability τ. To solve the maximum utility value of any node i, the utility function is:

(7) $$U_{i}=\tau \left(VS\prod_{j=1}^{n-1}\left(1-\tau \right)+VF\sum_{j=1}^{n-1}\left(\begin{array}{c} n-1\\ j \end{array}\right)\tau^{j}{\left(1-\tau \right)}^{n-1-j}\right)+VW\left(1-\tau \right)$$

The maximization condition is:

(8) $$\frac{\partial Ui}{\partial \tau }=VS{\left(1-\tau \right)}^{n+1}+VF\sum_{j=1}^{n-1}\tau^{j}{\left(1-\tau \right)}^{n-1-j}-VW=0$$

The best transmission probability of each node is:

(9) $$\tau^{*}=1-{\left(\frac{VW-VF}{VS-VF}\right)}^{\frac{1}{n-1}},i=1,2,\ldots$$

### 3.3. Date Transmission Stage

**Figure 4:**
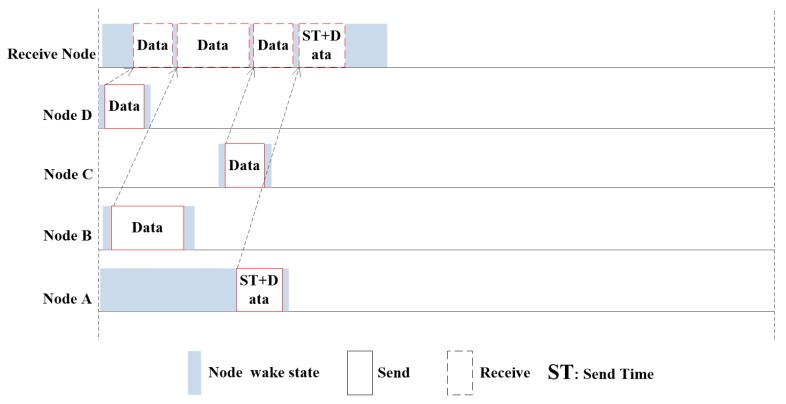
Data transmission process.

The receiving node exits the sleeping mode and enters the data transmission stage. The SN node transmits there date packet to the receiving node together with the sending table which contains the sending time of other nodes, and then enters the sleep state. The time of each node in the network is synchronous, so the sending node knows the specific time when the receiving node wakes up. All the sending nodes have a specific sending time. They wake up to send their own data packets at this specified time, and enter the sleep state after sending to reduce the energy consumption. After receiving the ST, the receiving node begins to accept the data from all other sending nodes that wake up according to the ST and start to send the data. In the end of the cycle, it will enter the sleep state again. When all the data in this cycle is received, the data upload is completed, and then it enters the sleep state again, waiting for the next cycle to wake up again. The details of the data receiving process are shown in Figure 4. The D node is first scheduled to send data when it enters the data transmission phase. Because of the spatial–temporal uncertainty in underwater acoustic network, the transmission time of other nodes may be the same or similar to that of node D. Even if the node B sends almost at the same time as node D, they will not collide at the receiving node, because the distance from node B to receiving node is longer than node D.

## 4. Experiment and Simulation

Based on NS2.3+Aqua-Sim simulation platform, we analyze the performance differences of DCN-MAC, SFAMA and ST-lohi. Both DCN-MAC and SFAMA protocols use the interaction of control packets to implement channel allocation. The difference is that the SFAMA protocol only completes a pair of nodes’ data transmission in each handshake. In DCN-MAC, the nodes exchange control packets to determine the transmission schedule, and once more handshake, the transmission of multiple data packets is achieved. The collision avoidance mechanism of DCN-MAC is similar to ST-lohi protocol. The ST-lohi protocol listens to the number of sending nodes in a period, then determines the back-off window period, and randomly sends contention channels during the back-off period. In the DCN-MAC, the scheduling node counts the number of nodes to be sent, and then obtains the information of multiple nodes in only one cycle to achieve scheduling. Compared with the larger backoff window of the ST-lohi protocol, it has higher efficiency.

### 4.1. Simulation Environment and Setting

**Figure 5:**
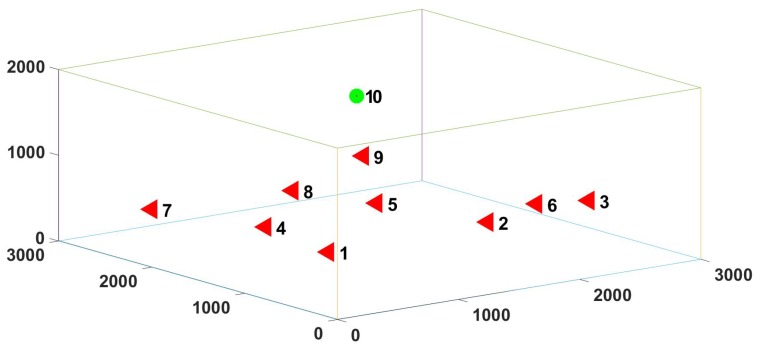
Topology of 10 nodes simulation network.

In the simulation experiment, we designed three different network scenarios, including 17, 9, 2 sending nodes and 1 receiving node, all nodes were within their respective communication range.The number of different nodes represented different network scenarios with different node densities. The nodes were distributed in a cube grid with the side length of 1000 m and side length ratio of 3×3×2. Figure 5 shows the network topology of 10 nodes. The distance between the sending node and the receiving node was no more than 3000 m. Each node was equipped with an underwater acoustic communicator according to the parameters of AquaSeNT [26]. The specific communication parameters are listed in Table 3. In the simulation experiment, we analyzed the throughput, delivery ratio, packet delay, and fairness of the three protocols. The definitions of the four parameters are shown as follows:

(10) $$\mathrm{Throughput}=\frac{\mathrm{Number}\;\mathrm{of}\;\mathrm{bits}\;\mathrm{received}}{\mathrm{In}\;\mathrm{time}\;T}$$

(11) $$\mathrm{Delivery}\;\mathrm{ratio}=\frac{\mathrm{Number}\;\mathrm{of}\;\mathrm{packets}\;\mathrm{received}}{\mathrm{Number}\;\mathrm{of}\;\mathrm{packets}\;\mathrm{generated}}$$

(12) $$\mathrm{Packet}\;\mathrm{Delay}=\frac{\sum (\mathrm{receiving}\;\mathrm{time}-\mathrm{generation}\;\mathrm{time})}{\mathrm{Number}\;\mathrm{of}\;\mathrm{packets}\;\mathrm{received},N}$$

(13) $$\mathrm{Fairness}\;\mathrm{Index}=\frac{\sum {\left(\mathrm{accepted}\;\mathrm{seq}-\mathrm{generation}\;\mathrm{seq}\right)}^{2}}{\sum_{1}^{N}{\left(N+1-2n\right)}^{2}}$$

### 4.2. Performance Evaluation

#### 4.2.1. Throughput

**Figure 6:**
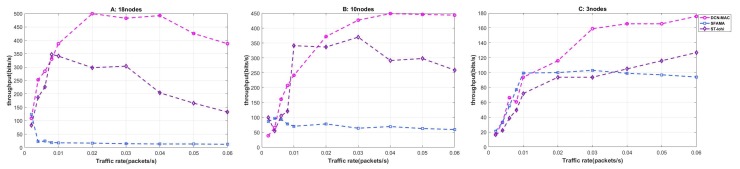
The throughput of the network at different packet generation rates when the number of nodes was 3, 10, and 18.

In this part, we mainly consider the network throughput under different network load, MAC protocol and node density. We compared the throughput of SFAMA, St LoHi and DCN-MAC in different node number environments with data packets generation rate. In the analysis, we defined the throughput as the number of bits transmitted by the network per unit time (BPS). The calculation method is shown in (10), and the simulation results are shown in Figure 6.

From the overall trend, the throughput of the three protocols increased first and then decreased with the growth of network load. This is because when the network load was too large, the protocols caused the throughput to drop because the number of packet conflict was increased and the nodes avoided more often.

Firstly, we analyzed the 18 nodes. When the node packet delivery rate was 0.002, the throughput performance of the three protocols was basically the same. However, when the packet generation rate rose a little, the performance of SFAMA protocol dropped sharply to only about 20 bps. It can be seen that the SFAMA protocol could not effectively handle the multi-node data conflict when the network load was high. This is because the SFAMA protocol needed to wait twice the maximum propagation delay of the network for each control packet transmission, thus causing more nodes to conflict. In addition, SFAMA protocol used the random avoidance of nodes to deal with conflict. It also introduced a large delay, which made the probability of conflicts increase again. The throughput of ST-lohi and DCN-MAC protocols was in the rising phase when the node’s packet delivery rate was in the phase of 0–0.01 packets per second, and the curves were basically consistent. However, when the packet delivery rate continued to rise, the throughput of ST-lohi protocol began to stabilize until the packet delivery rate was greater than 0.03. What is more, the throughput of DCN-MAC protocol declined until the packet delivery rate exceeds 0.04, and after the packet delivery rate exceeded 0.01, the throughput of DCN-MAC protocol was about 40% higher than that of ST-lohi protocol. The reason for this trend is that DCN-MAC protocol did not need all the nodes to avoid randomly according to the number of conflict nodes when dealing with multi-node conflict. It could schedule the data RTS of multi-node in a avoidance cycle. In contrast, when dealing with multi-node conflicts, the ST-lohi protocol required multiple nodes to perform the same avoidance algorithm according to the number of conflicting nodes N. It took at least N cycles to complete the processing of this conflict.

Then the throughput performance of the three protocols in different network scenarios was compared horizontally. In all three cases, the DCN-MAC protocol had the highest throughput, especially after network load increased, the DCN-MAC protocol performed better than the others. When the number of nodes was 10, the SFAMA protocol also showed a low throughput due to the huge network load and its own conflict avoidance mechanism. However, compared with 18 nodes, the throughput was increased to about 60 bps, because the overall network load was lower than the scenario of 18 nodes, so the throughput was improved. ST-lohi protocol and DCN-MAC protocol were similar in the scenario of 10 nodes and 18 nodes, and the trend was basically the same. Compared with 18 nodes, the maximum throughput of the two protocols was reduced by about 50 bps, and the degree of the priority in DCN-MAC protocol was reduced to about 20%. In the case of three nodes, it can be seen that the performance of the SFAMA protocol was significantly improved, and the throughput of the three protocols was basically the same when the packet delivery rate was less than 0.01. When the packet delivery rate was increased to 0.01, the throughput of SFAMA protocol tended to be stable at around 100 bps. However, the throughput of ST-lohi and DCN-MAC protocols continued to grow, and the change of DCN-MAC was more obvious than ST-lohi, and the performance of DCN-MAC was about 30% better than ST-lohi. The reason for this trend is that ST-lohi’s conflict avoidance mechanism still had a large probability of packet loss when dealing with multi-node conflicts, resulting in lower throughput.

In summary, the SFAMA protocol performed better than the other simulated protocols when the network load was low, and the protocol was relatively easy to implement. However, due to the fact that the SFAMA protocol could not effectively handle multi-node conflicts, so its performance decreased rapidly after the network load increased. Therefore, this protocol is more suitable for some network application scenarios where the number of nodes is low and the packet delivery rate is not high. The throughput performance of the ST-lohi protocol and the DCN-MAC protocol are basically the same at low network load, but the throughput of the DCN-MAC protocol is higher after the network load is further increased, which is about 35% higher than the ST-lohi protocol.

#### 4.2.2. Delivery Ratio

**Figure 7:**
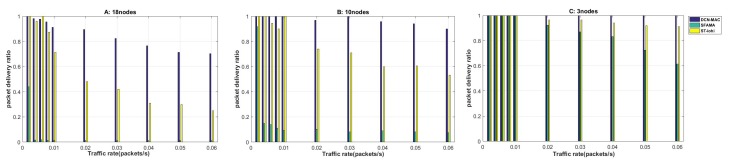
The delivery ratio of the network at different packet generation rates when the number of nodes is 3, 10, and 18.

The delivery ratio reflects the ability of the underwater acoustic network to process multi-node data packets. Generally, in the case of higher network load, the delivery ratio will be increased due to conflicts between data packets, which may result in the protocol failing to process these conflicting data packets in time, thereby reducing the delivery ratio. Therefore, the performance of delivery ratio directly reflects the conflict avoidance ability of underwater protocols. Here we define the delivery ratio as the number of packets successfully received by the receiving node compared to the total number of packets generated in the network. The calculation method is shown in (11), and the simulation results are shown in Figure 7. First, observe Figure 7 from a macro perspective. We can see that the trend of delivery ratio of the three protocols was consistent with the trend of throughput change in Figure 7. A high delivery ratio at the same packet delivery rate indicated a higher throughput of the corresponding protocol. This is because we calculate the throughput of the protocol based on the number of packets successfully received by the receiving node and the number of bits in the packet in a period of time. The delivery ratio of the MAC protocol was stable for a period of time as the network load increased, and then began to show a downward trend.

First, we analyzed the situation of 18 nodes. When the node’s packet delivery rate was 0.002, the delivery ratio of ST-lohi and DCN-MAC could reach 1, and the delivery ratio of SFAMA protocol was only about 0.4. When the data packet generation rate rose slightly, the performance of the SFAMA protocol dropped sharply to zero; that is, the data packets were basically lost at this time. This is because the SFAMA protocol needed to wait twice the maximum propagation delay of the network for each control packet transmission, and the nodes avoided randomly after the conflict occurred, thus causing more nodes to conflict. When the packet delivery rate was in the 0–0.01 phase, the delivery ratio of both ST-lohi and DCN-MAC protocols were basically stable below 1. When the packet delivery rate exceeded 0.01, the delivery ratio of the ST-lohi protocol began to decline first, and the rate of decline was always higher than the DCN-MAC protocol. In the process of decline, the delivery ratio of the DCN-MAC protocol maintained a 50% lead compared to ST-lohi. This trend indicates that the conflict avoidance mechanism of the ST-lohi protocol was not as robust as the mechanism of the DCN-MAC protocol. The DCN-MAC protocol performed better under the same network load. The reason for this trend is that the DCN-MAC protocol did not require all nodes to avoid randomly according to the number of conflicting nodes when dealing with multi-node conflicts. It could schedule data RTS for multiple nodes in an avoidance cycle. In contrast, the ST-lohi protocol required multiple nodes to perform the same avoidance algorithm according to the number of conflicting nodes N when dealing with multi-node conflicts. It took at least N cycles to complete the processing of this conflict.

Later, the delivery ratio of the three protocols in different network scenarios was compared horizontally. In all three cases, the delivery ratio of the DCN-MAC protocol remained the highest, especially after the network load increaseed, the DCN-MAC protocol performed better than the others. When the number of nodes was 10, the network load was still relatively high. Therefore, at this time, the SFAMA protocol also presented a lower delivery ratio because of its own conflict avoidance mechanism. However, compared with the results of 18 nodes, the delivery ratio obviously increased, and the stability was around 0.1. The ST-lohi and DCN-MAC protocols showed the same trend in 10 nodes and 18 nodes. The delivery ratio of the two protocols was 30% higher than that of the 18 nodes, and the performance lead of the DCN-MAC protocol was reduced to 30%. This performance is consistent with the simulation results of throughput performance. In the case of three nodes, we can see that the performance of the SFAMA protocol was significantly improved. When the packet delivery rate is less than 0.01, the delivery ratio of the three protocols is 1. At this time, the data packets in the network could be 100% processed. When the node’s packet delivery rate was increased to 0.01, and the delivery rate was reduced from the 100% delivery ratio at the packet delivery rate of 0.01 to the delivery ratio of 0.6 at the packet delivery rate of 0.06. The delivery ratio of the ST-lohi protocol also decreased, but the minimum has a delivery ratio of 0.9. The DCN-MAC protocol performed better, and the delivery ratio remained at 1 during the change in the packet delivery rate from 0 to 0.06.

In summary, the SFAMA protocol had a high delivery ratio when the network load was low, but the protocol could not be effectively processed the multi-node conflict. Therefore, the delivery ratio of the SFAMA protocol dropped rapidly after the network load increased. If the network load was too high, there may have been a case where the delivery ratio was zero. The delivery ratio of the ST-lohi protocol and the DCN-MAC protocol was basically the same at low network load, but the DCN-MAC protocol delivered a higher delivery ratio when the network load was further improved, with an average lead of around 35% of the ST-lohi protocol.

#### 4.2.3. Packet Delay

**Figure 8:**
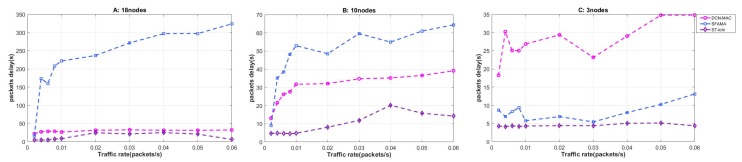
The packet delay of the network at different packet generation rates when the number of nodes was 3, 10, and 18.

Packet delay reflects the timeliness of network processing the node bursting data packet. The low packet delay means that the protocol handles the data faster, and the data processing is not delayed due to the mechanism of the protocol itself. In the underwater acoustic network, since the propagation delay of underwater acoustic communication is long, packet delay is an important performance to examine the underwater MAC protocol. In the simulation experiment, we defined the packet delay as: the time it took for a node to randomly generate a data packet to the node was served. The calculation method is shown in (12), and the simulation results are shown in Figure 8.

First, observe Figure 8 from a macro perspective. It can be seen that the packet delay of ST-lohi and DCN-MAC protocols was not directly related to the network load, and both of them did not show an intuitive tendency with the increase of network load. The packet delay of the SFAMA protocol increased as the network load increased. It had the same packet delay as the ST-lohi protocol in a low network load environment.

Studying at the three protocols separately, we can find that the packet delay of the DCN-MAC protocol in three different network scenarios was distributed around 30 s, which is closely related to the mechanism of this protocol, because the DCN-MAC protocol is a kind of periodic wake-up and sleep of the receiving node according to the duty ratio cycle mechanism. The receiving node slept for 50 s and then woke up for 10 s to start data receiving periodically. That is to say, the DCN-MAC protocol did not process the data packet quickly after the packet was generated, but waited until the receiving node woke up and uniformly received the data packet. Therefore, the packet delay of the DCN-MAC protocol was limited by the protocol mechanism and remained at around 30 s.

The packet delay of the ST-lohi protocol basically maintainsedat about 5 s when the network load was low, and it increased with the increase of the network load. This is because the protocol firstly divided the time slots when implementing the channel allocation, and the length of each time slot was T = τmax + Td, where Td is the time spent for processing the tone signal. In the ST-lohi protocol, the node to be sent firstly sent a tone signal to contend the channel. If the tone signal of other nodes was not received in the same time slot, it was considered that it occupied the channel and could start the transmission of data.

Therefore, in the case of low network load and no multi-node conflict, the packet delay of the ST-lohi protocol was the length of two time slots (about 5 s). When the network load was increased, the node was backed off, so the packet delay was improved.

The packet delay of the SFAMA protocol had a large relationship with the network load. This is because the SFAMA protocol had a high probability of node conflict when the network load was high. From having a node with a data RTS to successfully send and receive a data packet, it had to go through many conflicts and avoidance. Therefore, it took a long time, resulting in a delay up to 300 s under a high network load. In the case of low network load, the packet delay of the SFAMA protocol was stable at around 10 s. This is because SFAMA was based on the handshake to complete the channel assignment. Whenever a node had a data request to send, it sent an RTS control packet, requesting to occupy the channel to send the data packet. If the node received the reply CTS control packet from the receiving node, it meant that no other node contended for the channel at this time. The node started to send data, and in the end the receiving node replied with an acknowledge character (ACK) control packet to mark the completion of the current data transmission. Therefore, four times of packet transmission was required in this process, resulting in a packet delay of approximately 10 s.

#### 4.2.4. Fairness

**Figure 9:**
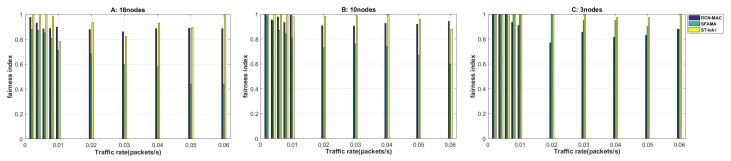
The fairness index of the network at different packet generation rates when the number of nodes is 3, 10, and 18.

The reason for examining the fairness performance is that the underwater acoustic channel has a long delay, and the node with a long distance will send the request first, but the node that is closer to the receiving node is processed first unfairly, due to the distance. The request acquisition of a distant node will not be processed, and the role of some sensor nodes is difficult to play. In the actual calculation, we calculate the fairness of the network by counting the data request order of the sending node and the service order of the receiving node. The calculation method is shown in (13), and the simulation results are shown in Figure 9.

Firstly, macroscopically observing the fairness results in the three network scenarios, we can find that the two protocols, SFAMA and ST-lohi, had strong links with the network load level, showing a trend of becoming fairer as the network load decreased. In addition, the fairness of the ST-lohi agreement always maintained a leading edge over SFAMA. The DCN-MAC protocol had consistent performance in different network loads, and the fairness of the network was stable at around 0.9.

The horizontal observation of the fairness of the SFAMA protocol in three scenarios showed that the fairness of the protocol varied widely among different network loads. SFAMA’s fairness could reach a maximum of 1, when the network load was small. When the network load increased, the fairness of the protocol was reduced to less than 0.5, and continued to decline. The fairness of the SFAMA protocol was so dependent on the degree of network load, which was caused by the mechanism of the protocol itself. The probability of conflict between nodes increased rapidly during high network load. The result of random avoidance caused the node data sent first to be post-processed or even lost, which seriously affected fairness.

The ST-lohi protocol was similar to the fairness trend of the SFAMA protocol, but the result was more than 20% ahead of the SFAMA protocol. It is because the ST-lohi protocol could make the sending node itself count the number of nodes that sent tone signals in the same time slot through the avoidance algorithm. It determined its own backoff behavior and did not need to wait to receive additional control packets from other nodes, which reduced the waiting time. Therefore, the multi-node conflict problem could be solved more efficiently, and the resulting unfairness between nodes was less.

The fairness of the DCN-MAC protocol was consistent in the three scenarios. When the network load was extremely low, the fairness was close to 1, but when the load was slightly increased, it stabilized at around 0.9. The reason why the fairness of the DCN-MAC protocol was low was not the conflict between multiple nodes, but the mechanism of the protocol itself. In order to further compress the total time used for receiving when calculating the sending table, there may have been a phenomenon that some nodes have data requests early but are serviced later. Therefore, the fair performance of the protocol was not optimal. In general, the fairness of the DCN-MAC protocol was limited by the mechanism of the protocol itself. It was difficult to achieve 100% fairness even in low network load, but it could reach 0.9 even at very high network loads. The SFAMA protocol had better network fairness only at low network load, and the fairness index was much lower than the DCN-MAC protocol and the ST-lohi protocol after the network load was increased. The fairness of performance of the ST-lohi protocol at high network load was similar to that of the DCN-MAC protocol, which led the DCN-MAC protocol by about 5% at low network load.

According to the simulation results: the advantage of the DCN-MAC protocol was that it could handle the conflicts generated during multi-node contention channels more efficiently. Moreover, the packet delay and fairness were basically stable, they did not drop sharply due to the increase of network load. Therefore, it had a greater advantage in the scenario of multi-node and high network load.

## 5. Conclusion

This article provides an DCN-MAC protocol for data aggregation and intra-cluster nodes to access scene scheduling. DCN-MAC is a MAC protocol initiated by the receiver to receive data in the form of data queues. This protocol takes into account the different characteristics of data packet length and propagation delay from different nodes to the receiving node. By strictly determining the sending time of the sending node, the data conflict between the nodes is avoided, the influence of spatial–temporal uncertainty is eliminated, and the spatial–temporal binary scheduling problem is solved. In addition, the protocol can adapt to the dynamic adjustment of the network structure, and the performance will not be reduced in the case of node traffic burst and topology change. In this protocol, the problem of multi-node conflict is regarded as non-cooperative incomplete information game, which can be solved by nash equilibrium equation and effectively deal with the state of multi-node wakes up at the same time. In the channel game, the conflict problem of multi-node is solved by one control packet interaction, which ensures the channel utilization when multi-node conflict. The simulation results based on NS2 show that the protocol has excellent conflict avoidance ability, can adapt to the dynamic network topology, moreover ensure stable and efficient throughput performance in a variety of network loads.However, this protocol is not suitable for networks that require immediate processing. Because this protocol uses a duty cycle mechanism, it has a high packet delay. Therefore, our main work in the future is to solve this defect and reduce the packet delay of DCN-MAC.

## Figures and Tables

**Table 1:** Game benefit of two nodes contend channels.

		Node j	
		Send	Wait
Node i	Send	VF,VF	VS,VW
	Wait	VW,VS	VW,VW

**Table 2:** Predigest game benefit of two nodes contend channels.

		Node j	
		Send	Wait
Node i	Send	−e,−e	VS,0
	Wait	0,VS	0,0

**Table 3:** Simulation parameters [26].

Parameters	Value
Communication radius	3000 m
Size of data packet	248 byte
Transmission power	10 w
Idle power	1 w
Communication rate	11,670 bps
